# A Review of Clinical Laboratory Education, Training and Progression: Historical Challenges, the Impact of COVID-19 and Future Considerations

**DOI:** 10.3389/bjbs.2023.11266

**Published:** 2023-04-12

**Authors:** Claudia Pearse, Sheri Scott

**Affiliations:** School of Science and Technology, Nottingham Trent University, Nottingham, United Kingdom

**Keywords:** COVID-19, education, training, clinical laboratory, biomedical scientist, professional development, HCPC registration, biomedical science and healthcare science

## Abstract

The COVID-19 pandemic had a wide global impact on society, including the clinical laboratory workforce. This historically underrepresented group of highly skilled professionals have now started to gain the attention they deserve. There had already been dramatic changes to laboratory training over the past 2 decades resulting from advances in technology, changes to service needs, and as a consequence of Pathology reform initiatives. The pandemic has had an additional impact. Higher education institutions and students adapted to emergency remote teaching. Clinical laboratories faced unprecedented challenges to meet COVID-19 testing demands and adjust to new ways of working whilst maintaining their usual high quality service provision. Training, assessment, and development arrangements had to convert to online platforms to maintain social distancing. The pandemic also had a global impact on mental health and wellbeing, further impacting learning/training. Despite these challenges, there have been many positive outcomes. This review highlights pre- and post-pandemic training and assessment for clinical laboratory professionals, with particular emphasis on Biomedical Scientists, outlining recent improvements among a history of challenges. There is increasing interest surrounding this vital workforce, accelerated thanks to the pandemic. This new public platform has emphasised the importance of quality diagnostic services in the patient pathway and in the response to national crises. The ability to maintain a quality service that is prepared for the future is grounded in the effective training and development of its staff. All of which can only be achieved with a workforce that is sustainable, invested in, and given a voice.

## Introduction

After years of global impact on society, the economy, health systems, education and working lives, COVID-19 no longer requires an introduction. However, the effects of the pandemic on clinical laboratory training and assessment have not been well documented. The impacts varied between the stage of career of the professional and the different pathology discipline. Undergraduates transitioned to online learning with fewer opportunities for basic laboratory experience. Post graduate laboratory professionals adapted to new ways of working and virtual methods of professional development as social distancing measures prevented activities such as conferences and the close shadowing of experienced peers. Workload varied significantly among different disciplines and staff were redeployed to meet service demands.

The global impact of COVID-19 and past pandemics on mental health and wellbeing has been well documented (1, 2), particularly in patient facing healthcare professionals such as doctors and nurses (3–7). There is also increasing research regarding the impact on students (8–11). However, there is limited data and published resources regarding the impact on laboratory professionals’ wellbeing (12, 13)—who are paramount to the patient pathway through diagnosis, prevention, treatment monitoring, and were central to the efforts against the pandemic. As poor mental health may impact learning and training, e.g., because of reduced motivation, concentration, engagement, and increased absence rates (14–18), this also falls within the remit of this review. In addition, reports of ‘long COVID’ are rising, including wellbeing symptoms such as ongoing fatigue, anxiety, depression, and brain fog (19–22), which may further impact an individuals’ ability to learn and develop professionally.

Several circumstances had altered the training requirements in clinical laboratories prior to the pandemic. This review aims to highlight the everchanging expectation of this workforce, as well as the further impact of the pandemic. Laboratory professionals and trainees have been instrumental in the pandemic and will be vital in future crises. Understanding and investing in their training and development is key, as we continue to rely on their readiness to provide quality patient care.

## Pre-Pandemic Laboratory Training and Assessment

### Pathology Networks

In the United Kingdom, increased NHS financial pressures, the emergence of the digital era, advances in technology, and the changing needs of healthcare providers and patients prompted the Carter Reports in 2006, 2008 and 2016 (23–25). The reports accelerated the transformation of pathology services into networks; with an emphasis on standardisation, digitisation and IT connectivity, and a more flexible workforce to improve efficiency, cost, and patient care (26). NHS England and NHS Improvement proposed a plan to create 29 Pathology networks in 2017, and as of 2019 they reported ∼97% engagement in networking from NHS Trusts (27). Thus, the clinical laboratory workforce had already been undergoing drastic change and uncertainty prior to the pandemic. Whilst the impact on cost and efficiency had received much attention; little has been investigated regarding the impact on laboratory staff (28). The Royal College of Pathologists (RCPath) published their own concerns about the Carter recommendations. Stating that consistent budget cuts seen by Pathology over previous years had resulted in a decline in workforce numbers through decreased retention and recruitment of laboratory professionals, an inability to fund locums, and the negative impacts on staff morale. Continued budget cuts could result in further consequences and hold serious implications for quality of practice. With some networks reporting a decrease in staff morale and retention, a loss of expertise, and negative impacts on training (29–31). A small 0.7% redundancy rate was reported in 2018 (28), however there were reports of increased early retirement due to mergers (30). Nevertheless, many networks have identified their mistakes, implemented changes to address them, and overall, Pathology networks are now receiving a more positive response (31). Although published research has not explored the impact on laboratory staff in detail, it has been noted that having larger networks will permit access to wider training and development opportunities for staff to expand their scope of practice and achieve increasingly senior roles. Furthermore, a more resilient and flexible workforce has been created, that can respond quickly to service user needs. For this to be realised, however, there is a requirement for continued investment and the process needs to be managed correctly (30–32).

### Modernising Scientific Careers

Following the Carter report (23, 24), the Modernising Scientific Careers (MSC) initiative arose in the United Kingdom in 2008 (26). MSC aimed to standardise education and training for healthcare science professionals; building a clear career framework with flexible routes of entry and progression that would attract and retain staff, ensuring training was fit for purpose, and improving value for money from diagnostics. Within the proposed framework, MSC acknowledged that the advances in technology and the changing needs of service users meant appropriate education, training, and continual professional development (CPD) opportunities would be vital in coordinating a flexible workforce capable of the high level of care expected (33, 34). Despite being awarded the Guardian Workforce Innovation award in 2013, the initiative received criticism from the workforce. Although the Scientist Training Programme is in full swing, the Healthcare Science Associate and Practitioner pathways have declined in popularity and availability as standalone degree programs (35, 36). The term “Healthcare Science Practitioner” (HCSP) in itself has caused confusion, as these professionals identify as Biomedical Scientists and are registered as Biomedical Scientists under the Health and Care Professions Council (HCPC), with a professional body called the Institute of Biomedical Science (IBMS). Consequently, the level 6 Healthcare Science Practitioner apprenticeship, is now under review, with the development of a standalone level 6 Biomedical Scientist standard soon to be released.

### Training and Assessment Requirements of Laboratory Professionals

**FIGURE 1:**
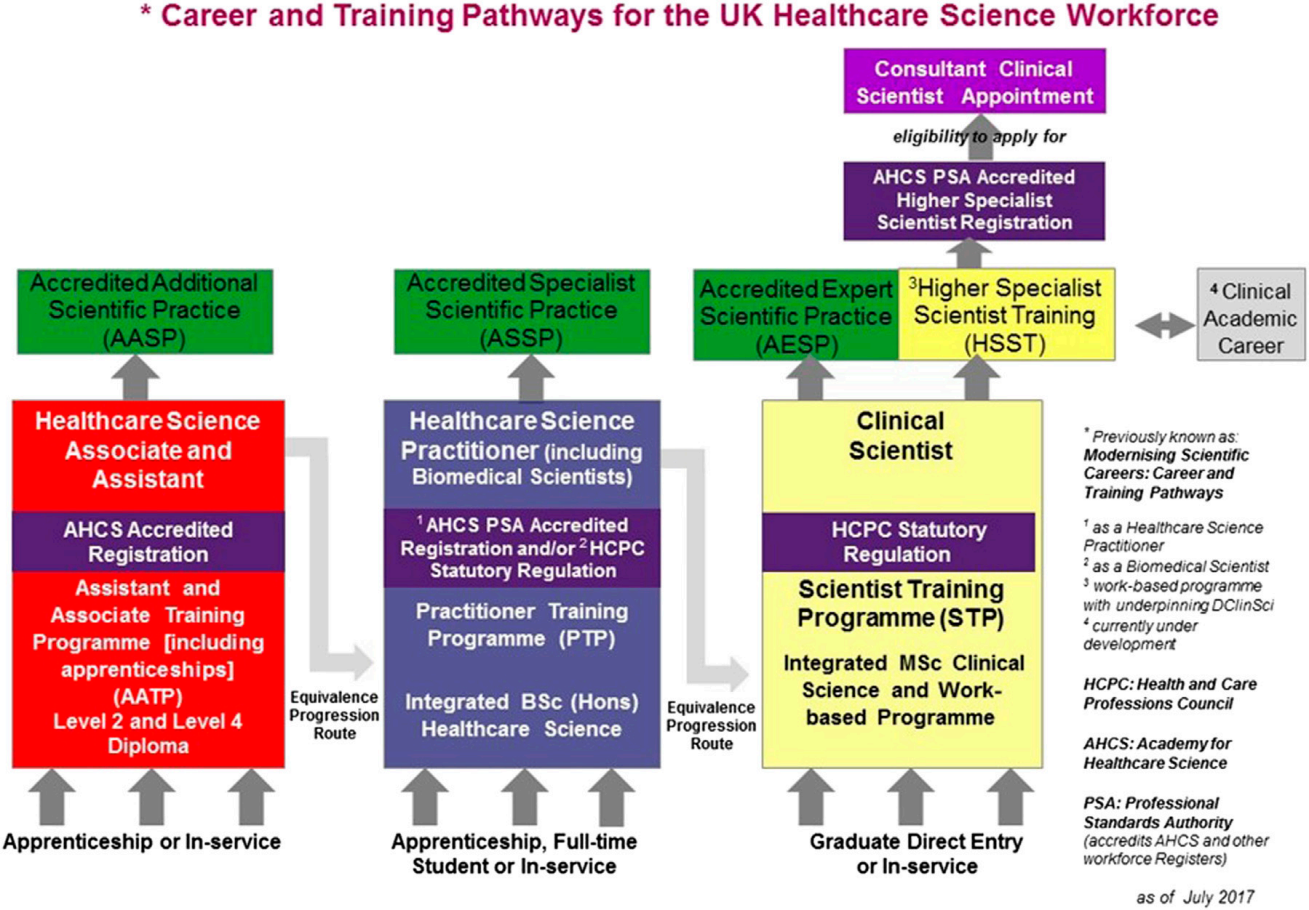
Taken with permission from The National School of Healthcare Science and the AHCS. Showing the NHS England Modernising Scientific Careers framework with adaptations as agreed by the AHCS. Including pathways for support staff (Healthcare Science Assistants/Associates; HSAs), Biomedical Scientists/Healthcare Science Practitioners (HCSPs), and Clinical Scientists. There are various entry routes, these include: apprenticeships, undergraduate, graduate, in service, and equivalence progression routes (40).

The clinical laboratory workforce is diverse and there are various routes of entry and progression that differ with respect to the Pathology discipline. To understand the impact the pandemic has had on training and assessment, some understanding of the career framework is necessary. For those less familiar, a summary of common routes of entry to the HCPC register in the UK are provided in Figure 1 but this is not exhaustive. The IBMS and the Academy for Healthcare Science (AHCS) are two Professional Bodies which provide accreditation of the educational programs, provide a training and assessment model for registration, and support CPD of clinical laboratory professionals in the United Kingdom. The professional bodies work in tandem with the regulatory body, such as the HCPC, who set the standards for registration. This ensures a robust training and assessment program to promote safe and quality practice. Registered laboratory professionals have a mandated requirement for CPD and lifelong learning. This ensures registrants remain competent to provide a safe, lawful, and effective service. This is emphasised and regulated by the HCPC in line with their standards of proficiency. Registrants are called upon at random during registration renewal periods to provide evidence that their practice continues to meet the standards of proficiency, including documentation of regular CPD activities. Failure to evidence CPD can result in removal from the register (37–39).

**FIGURE 2:**
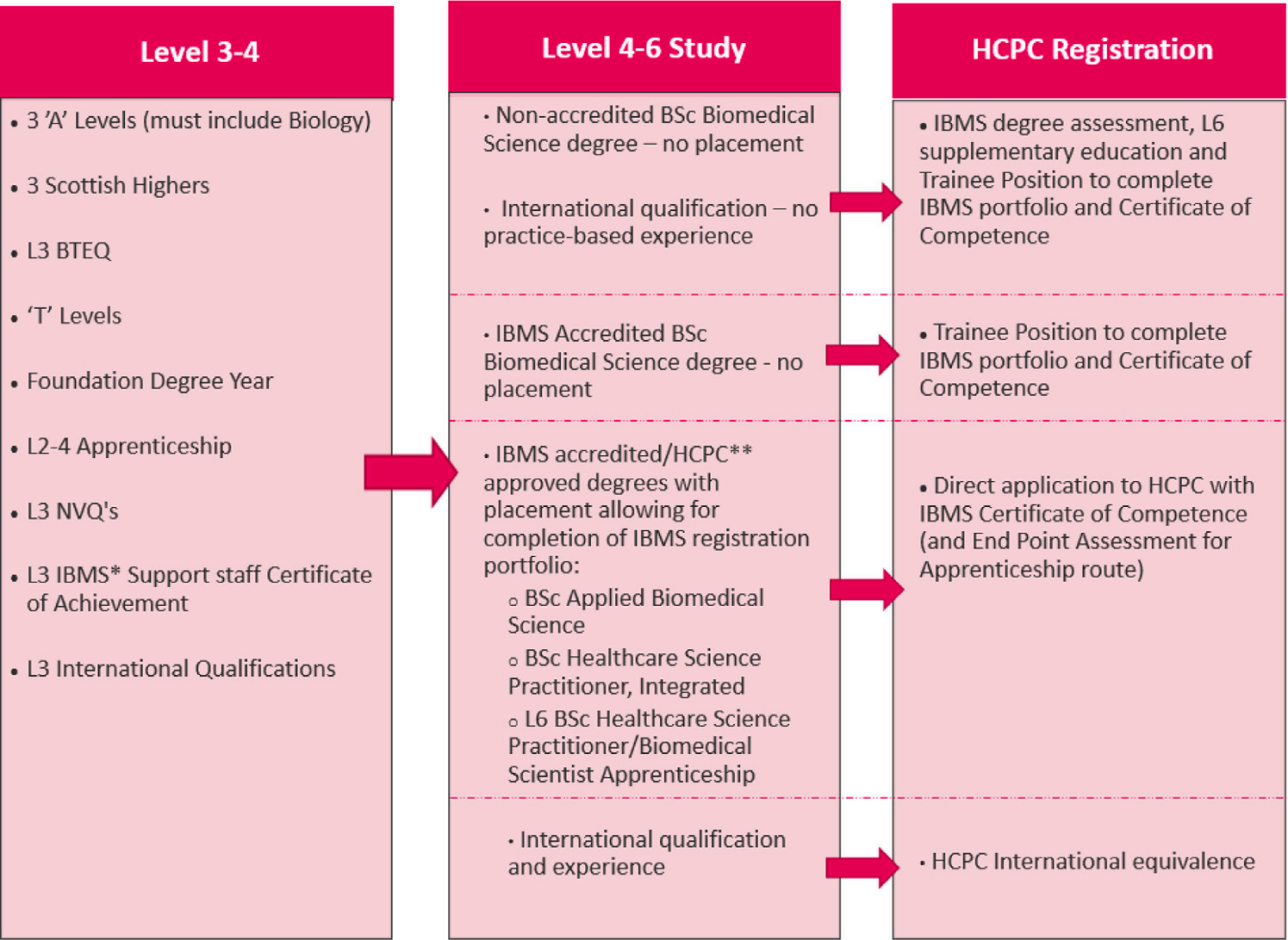
Summary of routes to HCPC registration as a biomedical scientist. *Institute of Biomedical Science **Health and Care Professions Council.

For entry level to Biomedical Scientist posts in the UK, potential applicants must be registered with the HCPC. Trainees require both sufficient level 6 knowledge and practice-based competency (41, 42). This is achieved through a work-based competency portfolio, the IBMS registration portfolio, to achieve the IBMS Certificate of Competence (IBMS CoC). As such, trainees require laboratory work-experience in an IBMS approved training laboratory either during their degree or after. The portfolio can be completed through applied courses with a sandwich/integrated placement or *via* an apprenticeship route. It is worth noting that opportunities are limited in comparison to full-time courses. For a summary of entry routes to HCPC Biomedical Scientist registration please refer to Figure 2. International applicants may also be accepted on to the HCPC register as Biomedical Scientists through an equivalence assessment pathway.

The verification process for the IBMS CoC consists of a laboratory visit by a trained verifier. The verifier assesses the portfolio to ensure HCPC standards have been met and the trainee provides a commentated tour of the laboratory. Biomedical Scientists can progress further in their role with postgraduate qualifications provided by higher education institutions (HEIs) and/or professional bodies, through qualifications such as the IBMS Specialist and Higher Specialist portfolios.

HCPC registration as a Clinical Scientist can be achieved through HCPC approved programmes including the NHS Scientist Training Programme facilitated by the National School of Healthcare Science, the AHCS Certificate of Equivalence, Association of Clinical Scientists (ACS) Certificate of Attainment, IBMS Certificate of Attainment in the case of Clinical Biochemistry, Clinical Immunology, Haematology and Clinical Microbiology, and also the HCPC International Route (43, 44).

## Post-Pandemic Laboratory Training and Assessment

### Quality of Teaching and Learning

As the pandemic became a major global health concern with increasing cases and mortality, intermittent lockdowns and social distancing took effect and universities had to rapidly transition to remote teaching to support the progression of their students. Online learning and blended-learning approaches increased in popularity over the years, due to advances in information communication technologies (ICT), increasing class sizes, changes to student needs and expectations, the emergence of Massive Open Online Courses (MOOC) and a drive to improve the quality of teaching and learning (11, 45, 46, 47). Research has indicated that if properly executed, online learning has many potential benefits (48, 49). Including promoting self-efficiency and the skills necessary for lifelong learning (50), which are key requirements of laboratory professionals working in ever changing healthcare environments. However, online learning requires motivation and self-discipline, thus inexperienced learners may need additional support to learn independently and may benefit from synchronous activities, whereas older, experienced learners may benefit from the flexibility of asynchronous styles that more easily accommodate personal and working lives (51–53). There are many other variables that complicate online delivery, emphasised by the development of frameworks that aim to support programme development. Examples include the Community of Inquiry (CoI), and the Technological Pedagogical Content Knowledge (TPACK) models (46, 54–56). Recent reports have sought to clarify the important difference between a well-developed online course and temporary “Emergency Remote Teaching” (ERT). Making this distinction when assessing the global impact of the pandemic on teaching and learning will be necessary to draw meaningful conclusions and reduce the risk of undermining well-established online approaches (46, 51, 57, 58). For example, based on the CoI framework; social, cognitive and teacher “presence” are three components important in creating an online community capable of deeper-level thinking and critical reflection. Social presence does not develop organically or quickly online, as students are physically separated from peers and educators. To combat this, tools to achieve social presence are consciously embedded into course design to promote online collaborative learning (54, 55). This was something there may have been little time to consider in the rapid shift to ERT. Nevertheless, the pandemic has resulted in the acceleration of an established notion that an increase in online learning provision can meet the market demands of the 21st century. The pandemic has highlighted challenges that can be used as opportunities for HEIs. Having a greater online presence with effective course design will help to serve a wider population of students, ensure preparedness for future disasters, and help maintain market competitiveness as growing numbers of students and professionals seek flexible learning opportunities. This should encourage HEIs to invest in digital technology and staff development to improve teaching and learning (11, 46, 47, 50, 58, 59).

There are several studies that aim to evaluate the impact of ERT on higher education students, and conflicting results have been published (11, 58–61). Key challenges include internet connectivity, use of technology, home life distractions, lack of social interactions, difficulties in concentration, lack of motivation and mental wellbeing consequences (60). The effects of online teaching in general are difficult to measure, as it is a unique experience to the learner with many variables, including course design, interaction with peers/teachers, class size, subject discipline, international/home student, age, gender, ethnicity, personal and work life, digital competency, access to technology and internet connectivity (51, 58, 60). Furthermore, the impact of ERT is being evaluated with different objectives. Including student/teacher perceptions, academic success based on assessment outcomes, student’s preparedness for future study/work, and engagement. ERT approaches also differed, influenced by variations in staff digital competency, knowledge of e-pedagogy and resource availability. When narrowing down the search to Bioscience courses, results have also varied. A UK study found out of 151 undergraduate Bioscience students surveyed, open book online exams were preferred, and exam performance improved compared to pre-pandemic figures. This study suggests that there was an increase in engagement, as students found it easier to communicate *via* chat functions. However, there was reluctance to use cameras in live sessions. Students reporting concentration difficulties increased by 39% compared to pre-pandemic survey results but there were minimal concerns over using technology. However, findings of a lack of motivation, household disruptions, and poor internet connectivity were common challenges. In addition, reports of an inadequate working space indicated inequality among students (11). This was further supported by a survey of 75 Bioscience students in Greece which also found using technology was of no concern, but engagement was decreased, and students were uncomfortable turning on their microphones. Students enjoyed the flexibility and convenience of remote learning, however missed the social interaction with peers and faculty and expressed concerns about missing laboratory practicals (59). A study in Malaysia which surveyed 120 undergraduate Biological Science students found students were generally satisfied with their online learning experiences but were also concerned about the lack of practical laboratory sessions. Students reported lower engagement with peers and faculty and found group work difficult as social bonds had not been formed and motivation differed between peers. Unlike the previous two studies (11, 59) students did report difficulties in using technology, in addition to poor internet connectivity. Further challenges included home distractions and time zones being a challenge for international students to attend live classes (61). A study in Malaysia of 112 undergraduate Bioscience students aimed to assess student perceptions. Most respondents found they had increased flexibility. However, 68.8% reported challenges in effective learning, with 45.5% reporting internet issues had a negative impact. When asked about preferred modality, consensus was split between a hybrid approach and face to face, with <2% preferring completely online delivery (62).

**FIGURE 3:**
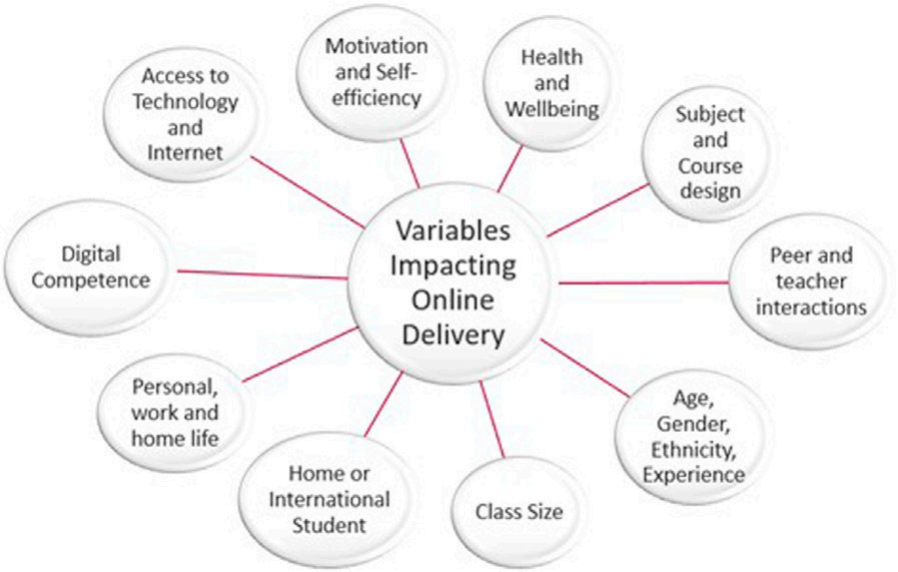
Summary of variables that impact online course delivery and the Learner’s experience.

Nevertheless, small sample sizes and multiple variables makes it difficult to draw conclusions from such studies and further research is required. In addition to the variables that impact online delivery (Figure 3), the geographical impact of the pandemic at any one time varied. Differences in spread, morbidity, mortality, government measures and lockdowns between regions and countries add another level of complexity to understanding the impact on education. In addition, inequality in HEI resources, staff digital competency, the different survey/experimental designs used to assess impact, and how and when engagement, perceptions and learning outcomes were measured will alter research findings. Considering the variables that constitute the learners experience, research within HEI departments should aim to identify the specific needs of their students to personalise learning and share best practice (63).

### Online Assessments and Exams

Many HEIs also moved assessments and exams to fully online formats. Universities had to design assessments and exams based on higher-level thinking, application of learning and reasoning, rather than retention and recall to effectively assess knowledge and understanding in open-book and multiple-choice assessments (50). Students were given extended time frames to complete exams to account for time differences, childcare/caring responsibilities, religious commitments, internet connectivity, etc. The transition of exams from conventional lecture halls to online has been well received by students, and some studies report academic achievement has not been negatively affected (11, 50). However, concerns of academic malpractice were heightened (46, 50, 54). Furthermore, difficulties were seen as life returned to “normal” and students either lacked the skills required for on campus assessments or experienced heightened anxiety over these types of assessment. An increase in students becoming increasingly stressed and anxious about readjusting their revision and the mind set required to prepare for timed, on-campus exams became noticeable throughout HEIs. This was particularly increased for students who started university during the pandemic, and thus had only experienced online formats. This precipitated the requirement for increased support to facilitate the change (60).

### Virtual Verifications

IBMS assessments also moved to virtual formats. Registration and specialist portfolios were digitised and sent to the verifier or examiner, respectively, to be reviewed before a virtual meeting. Laboratory tours took a variety of methods, e.g., PowerPoint presentations, pre-recorded videos, and live streams. The IBMS took a flexible approach, allowing creativity for laboratory tours and use of multiple ICT platforms such as MS Teams, Zoom, and Skype. With the appropriate platform determined by the parties involved. The IBMS supplied templates for constructing a digital portfolio but urged assessors to be flexible with their approach to assessing these, prioritising fulfilled HCPC requirements over ePortfolio format (64). To maintain equality and standardisation of the verification process, advice was provided regarding the 90-min time allowance, stating that this should remain for the review of the portfolio, even though portfolios were received prior to the online meeting. The implementation of virtual assessments received positive feedback from both verifiers and assessors. Receiving the portfolio before the meeting permitted missing/supplementary information to be requested and sent before the formal meeting and tour, streamlining the process and avoiding trainees having to send further documents after their assessment and anxiously wait for the verdict (65). Furthermore, in 2020, the IBMS reported an increase in the number of volunteers stepping forward to do assessments following the transition to virtual methods, as geographical challenges were overcome (64), and this may have benefitted trainees in rural laboratories. However, Healthcare firewalls/IT restrictions can make sending large, digital portfolios difficult, and can cause issues with opening documents. Difficulties with virtual laboratory tours included internet/phone connection, risks of filming confidential information, and laboratory health and safety barriers (no mobile phones should be taken into the laboratory). Electronic signatures also caused concerns over authenticity, and IT around authenticating signatures was not well understood by all (65). The volume of evidence for the specialist portfolio caused particular difficulties in evidence transfer.

The IBMS has an online platform for CPD modules, although at the time of this review it is not currently used for registration/specialist assessments, the IBMS aim to move these assessments to an eLearning platform in the future. Thus, the portfolio evidence will be more easily accessed by all parties, which will standardise the process and circumvent some of the current difficulties (65). The IBMS are keen to maintain virtual verifications, especially where verifiers/assessors are not local. As the combination of a steady increase in trainees wishing to achieve HCPC registration or progress within specialist areas over the years, and difficulties in obtaining assessors and examiners that are willing or able to complete assessments have resulted in many trainees experiencing delays in their route to registration/development. It is worth noting that the position of verifier/examiner is currently voluntary, requires training from an IBMS representative, and is mostly carried out by IBMS members as CPD in line with their willingness to support training. The current version of the IBMS registration portfolio is also due to be amended following recent additions and amendments to the HCPC standards for Biomedical Scientists, summarised in Table 1 (66), effective from September 2023. The results of these reforms may further improve the training process.

**TABLE 1:** Changes to biomedical scientist HCPC^a^ standards of proficiency 2023.

Wording changes to include “Must” and/or “Take Action” to ensure movement away from passive understanding of professional requirements towards an active implementation
Inclusion of the promotion of public health and preventing ill-health
The expansion of Equality, Diversity and Inclusion
Centralisation of the role of the service user
Increased emphasis on registrants in looking after their own mental health
Increased emphasis on digital skills and currency
Increased emphasis on the role of leadership

^a^ Health and Care Professions Council.

### Practical Skills

Bioscience students, and other practical courses, are in a unique position. The nature of Biomedical Science requires both an understanding of theory, and competence in practical techniques and professional practice (1,167). This is emphasised in The Quality Assurance Agency for Higher Education (QAA) benchmark for Biomedical Sciences degrees (68), and in the IBMS requirements for accreditation of BSc Biomedical Science degrees (42), which includes a mandatory research project. Pandemic restrictions led to closure of many HEI laboratory facilities. Meaning the opportunity for students to gain practical skills was severely diminished (11, 49). To address this issue, HEIs increased the use of virtual technologies. Examples included commercial laboratory simulations such as Labster and LearnSci (69, 70), HEI recorded videos of staff completing practicals with attached activities, and experiment kits were sent to students to complete in their own homes (63, 71, 72). However, challenges to moving practical classes online differ depending on HEI resources, staff training, and access to technology (71). Not every student had access to a smart phone, laptop, or a good internet connection, and HEIs did not always have the funding to invest in advanced technology infrastructures or take-home experiment kits (63). For those unable to afford commercial laboratory simulations, there were several open access resources available (49, 73) such as LabXchange (72).

There are many advantages to online laboratory sessions, and they have been shown to complement practical sessions well, to improve teaching and learning outcomes by encouraging active learning, prior to the pandemic (49, 74, 75). Online pre-laboratory sessions can help to familiarise students with the experiment and equipment to improve understanding and confidence in performing the practical. This then is followed by an online post-laboratory session to consolidate learning (49, 74). However, the skills gained from the hands-on experience with equipment, working with others, and following good laboratory practice procedures cannot be gained completely by online methods (49, 58, 74). This was emphasised by the guidance from the QAA (76) regarding practical sessions during the pandemic, which allowed for streamlining the requirements as not to delay student progression. The QAA suggested alternative online practical sessions that allowed data interpretation and problem solving, e.g., by providing students with a video of an experiment being conducted, and providing the data generated from previous experiments for them to analyse and interpret. They suggested further that learning outcomes that were assessed, through sessions with outcomes unachievable by a remote alternative, e.g., competence in handling equipment, should be postponed until restrictions lifted to allow them to take place. However, it was further stated that if those learning outcomes have already been achieved in previous laboratory sessions/modules, they could be disregarded (76). In consequence, some universities organised additional laboratory practical sessions post lockdown to bridge any perceived gap (61). Universities realised the benefits to online learning providing flexibility to students and faculty (50, 59, 61) but also recognised the importance of maintaining social interactions and practical skills. Thus, in some circumstances a hybrid approach would be more appropriate to ensure graduates have the skills required for industry, whilst using digital practical sessions to complement learning and improve engagement (11, 62, 74, 75).

Even with virtual simulation, it can be argued that the advanced clinical laboratory workplace skill acquisition cannot be well replicated within a HEI setting. With increasingly automated testing in clinical laboratories, the use of large multi-channel analytical platforms and advances in digital technologies, there is less of an emphasis on conventional discipline specific tasks and manual methods. Instead, there is a need for a flexible, cross-disciplinary workforce with broad skills mix to allow to adapt to service needs. Thus, vital skills now required of graduates may include an awareness of automation and analytical platforms, research and innovation, leadership, digital and ICT competency, method validation, quality assurance and bioinformatics (71, 77, 78). It has been suggested that transforming the curriculum to support the development of such skills is possible through online learning and may better prepare Bioscience graduates for industry needs (71), rather than just those handful that are able to secure one of the limited placement opportunities. Thus, the pandemic may have presented an opportunity to revise and adapt current curriculum to reflect service needs whilst reducing the strain on HEI laboratory resources. Some on campus laboratory classes could be replaced with data analysis projects that promote bioinformatics, problem-solving, and interpretation skills (79) that are highly appliable to many disciplines in biomedical science today. This could also offer increased flexibility and a greater choice to students in their final year research endeavours, which can improve their motivation and engagement (77). Indeed, the Royal Society of Biology (RSB) recognised this prior to the pandemic as evidenced in their updated accreditation requirements (79, 80). The pandemic accelerated the adoption of such strategies by the IBMS, who have published some examples of final year projects that can be carried out remotely and fulfil the IBMS accreditation requirement for Biomedical Science degrees (71, 81). Off-site “dry” research projects also present an opportunity for collaboration between HEIs, students, and industry and a chance to expand placement provision, usually limited by industry resources and willingness (82). Benefits include a wider choice of project types, an understanding of industry needs, and improved employability for students. HEIs can form valuable links with industry and keep up to date with current practice which will enhance teaching. Finally, industry partners can benefit from expanding their pool of appropriately skilled graduates and welcome the introduction of new ideas and innovations into their organisation (71). Projects can be designed around industry needs to have a real-life impact. Although some examples of this are already in place, they are limited. The pandemic has highlighted their potential and as a possible solution for tackling the lack of placement opportunities for students (71). One example of such a venture (83) created a digital internship for Microbiology students that enabled collaboration, research skills, communication, scientific literacy, and digital competency. Students worked remotely with peers in a team, supervised by the faculty, to annotate a series of Microbiology podcasts discussing the latest research and techniques from primary sources. Students were tasked with aligning the podcast content to the professional standards and curriculum learning outcomes, while annotating them accordingly. In addition to the plethora of skills achieved by the students, the internship created work experience opportunities, improved course engagement, and created valuable education resources for teaching (83).

Virtual technologies such a virtual reality, augmented reality, and serious gamification may be a good addition to course programmes to better prepare students for industry, providing they remain up to date and relevant. Such methods can immerse students in a virtual, yet real life workplace scenario, where they can make mistakes and learn from them without causing harm to peers or patients (46, 67). Applications could be tailored to recreate clinical laboratory situations that cannot be well replicated in HEI facilities, e.g., prioritising urgent samples or preventing pre-analytical variables such as incorrect blood tube use, as demonstrated in a recent study (67). Although, more research on the effectiveness of digital simulations for laboratory training are warranted (82), there are clear benefits to incorporating them into teaching practices to improve student understanding and engagement in practical sessions (74, 75).

Laboratory placements are limited to what can be supported by clinical partners and apprenticeship provisions (67, 82), limiting access to the experience required for HCPC registration. It has been stated that the impact of the pandemic on Bioscience placements/internships is not well known but is worthy of attention (71). The pandemic may have decreased opportunities because of social distancing measures and the increase in remote working. In contrast, it may have provided new opportunities for online collaboration (71, 83) and increased the scope for a different kind of laboratory experience. For example, in response to the pandemic, the HCPC and IBMS worked with the UK Government to arrange a temporary HCPC register for some healthcare professionals to increase staffing resources. This register allowed former registrants (left within 3 years) and final year students (on accredited courses) to temporarily practice under protected titles, therefore providing students with the opportunity to be part of the pandemic efforts. However, the uptake of temporary registrants into the workforce was low at 10% (84). Nevertheless, following closure of the temporary register, 37 Biomedical Scientist and Clinical Scientist students that entered practice were kept on the register as they progressed towards achieving permanent registration (84). A further example of the pandemic generating workplace experience opportunities includes Derby University students involved in COVID-19 testing centres and receiving encouragement to become volunteer vaccinators (85). As diagnostics become more patient focussed and there is a drive towards increased use of point of care testing (86, 87), such experience is valuable to future trends in Biomedical Science (78) and helps to reiterate to students that there is a patient at the end of every sample. In addition, the pandemic accelerated open online learning resources, e.g., *via* social media and podcasts, which extended real industry knowledge to scientific, student/trainee, and public communities. As conferences moved online, education and professional development opportunities became more accessible than ever before. A particular benefit to trainees in rural areas who may usually struggle to access resources due to geographical constraints and the expense involved in travelling long distances, not to mention the sustainability benefits if this trend continues going forward.

### The Impact of the Pandemic on Clinical Laboratories

There is very little published information detailing the impact of the pandemic on the clinical laboratory workforce (12, 13, 87). Lee et al (12) examined the psychological impact of the pandemic on laboratory staff, which is discussed later in this review. From the limited data available however, it’s apparent that the effects of the pandemic were felt differently between pathology disciplines. Microbiology/Virology took the brunt of the pandemic testing initiatives; validating and implementing new COVID-19 tests and changing working practices to meet the large demand, with many laboratories converting to a 24/7 rota. Whilst other disciplines may have experienced a dramatic decrease in workload due to the cancellation of routine and non-urgent appointments, surgeries, and testing. The decrease in workload provided fewer opportunities for staff training and development, as samples were limited to urgent only requests (89, 90). Lucey and O’Connor (13) conducted a study in Ireland, where they surveyed 272 laboratory professionals at various grades across all major disciplines. Impacts reported by respondents included an increase in working hours (48%), and increased complexity in the types of tests being performed (70%). The impacts of the pandemic can be found summarised in Tables 2, 3. Interestingly, professionals working in multidisciplinary and Biochemistry laboratories reported a significantly greater number of additional hours worked when compared to staff in Microbiology. A possible explanation for this could be the reduction in workforce due to isolation of the vulnerable, sickness and/or an increase in workload through an increase in hospital admissions. This is contradictory to a study in Pakistan that surveyed 50 Clinical Chemistry laboratory professionals and found 80% reported increased ability to maintain accreditation and quality standards as their workload decreased from the decline in non-urgent testing. These are tasks which usually cause stress and are time dependant heavy (88). Notably, the study had a small sample size and focussed solely on one discipline with no comparisons between disciplines. Lucey and O’Connor (13) go further, explaining Microbiology reported the most significant rates of change in rota schedules and higher complexity in the types of tests they were performing in comparison to other major disciplines. The authors attribute this to the implementation and validation of molecular COVID-19 testing platforms, of which a variety were needed to manage the fluctuating availability of reagents and consumables. In addition, 21% of respondents reported disruption to training and this occurred at all levels; undergraduate, Masters, PhD, CPD and professional development activities. However, positive reports included staff feeling a sense of pride for the work they were doing and acknowledging the importance of collaboration to meet service needs, as well as their ability to adapt to new ways of working (13). Many staff working in disciplines that experienced a decrease in workload were retrained and redeployed to areas of need, e.g., to support sample collection, processing, and testing (89). Further emphasising the need for a flexible workforce and training that is fit for purpose to allow staff to respond to crises. In addition, staffing levels fluctuated as the vulnerable and those testing positive isolated, and those with childcare responsibilities were unable to attend following school closures (90). As restrictions have now largely lifted, new challenges arise. There is a large backlog of tests as the NHS catches up on postponed appointments and surgeries (91). RCPath have expressed concerns over cancer diagnostics in Histology and Haematology, and in Blood Transfusion provision. Stating the workforce is currently understaffed to deal with the backlog and the increase in chemotherapy is likely to increase the requirement for compatible blood. Further added concerns include blood donations which were stifled due to restrictions and illness (92). However, one hopeful outcome of the pandemic is that such workforce challenges were highlighted and warranted increased funding from the government’s COVID-19 response group. In an IBMS online Webinar discussing developing the scientific workforce (93), Ruth Thomsen (Scientific Director, NHS England) introduces the implementation of Practice Educators into laboratory networks across London thanks to such funding. These Practice Educators are tasked with identifying workforce challenges, including training, development, and progression. They are working across laboratory networks to identify key barriers and skills gaps and share best practice between regions. This new role and collaborative effort in identifying challenges and areas for enhancement will provide key insights and data that may guide improvements to training, development, and service provision for the future.

**TABLE 2:** Summary of negative COVID-19 impacts on course and training delivery.

Rapid transition to Emergency Remote Teaching without thorough course design
Practical work and technical skill development limited
Online assessments and exams, and increased stress and anxiety as these returned to campus
Inequality in Higher Education Institution resources and staff/student digital competency
Online interactions only, reduced social interaction and ability to form bonds
Reduced motivation and engagement
Time zone differences and restricted overseas travel due to lockdowns impacted international students
Personal, work and home life challenges, e.g.,
• nursery/primary school closures impacted childcare
• increased home distractions and difficulties concentrating
• financial circumstances, e.g., reduced hospitality sector
• increased workload and altered ways of working for many hospital staff/apprentices/placement students
Lockdowns limited access to workspace, technology and internet
Mental health and wellbeing compounded by isolation, risk of infection, finances and concerns over loved ones
Disruption to Clinical Laboratory Training as COVID-19 efforts prioritized

**TABLE 3:** Summary of positive COVID-19 impacts on course and training delivery.

Innovative curriculum and revision of healthcare service needs
Potential expansion of project and placement types and provision, e.g., dry data analysis and digital internships
Improved digital technology and competency (utilisation of virtual conferencing tools, e.g., Microsoft Teams/Zoom)
Online verifications and conferences; education and professional development opportunities more accessible and sustainable
Acknowledgement of the importance of Biomedical Science in patient care
Importance of Training and Continual Professional Development highlighted, required to maintain an adaptable and flexible workforce that can meet service user needs and are prepared for future crises
Workforce challenges highlighted leading to increased funding and planning
Greater emphasis on supporting mental health

### In the Eyes of the Public

Possibly the largest Impact of the pandemic on the workforce was that the role and importance of biomedical science gained public attention (87). Pathology, encompassing all disciplines, has gone through substantial transformation, and has faced many budget cutting initiatives throughout the years (29). Despite being involved in 70%–80% of patient care pathways, Pathology is underfunded and has had little representation when NHS budgets are being delegated (23). The lack of wider understanding regarding this workforce’s contribution can be seen by the frustration in laboratory staff from media misconceptions regarding who is performing the COVID-19 tests, as these professionals felt unrecognised for their contribution to testing (13). In addition, during the early stages of the pandemic, despite all the work that has gone in to developing pathology networks with good links and communication, the government opted to invest in separate ‘light house labs’ to increase COVID-19 testing capacity to meet their growing testing targets (94). These laboratories came across several challenges that demonstrate the complexities involved in running a quality assured, fit for purpose, laboratory network. For example, the IT systems were not able to transmit results to NHS patient records (95), defeating the purpose of the ‘test and trace’ efforts to prevent the virus spreading. Considering standardisation and developing IT connectivity were key aspects in the move to develop pathology networks, as discussed above. If this established, knowledgeable workforce had been given more of a voice early in the pandemic, policymakers may have been able to make better informed decisions on how to boost testing capacity. Furthermore, the rapid implementation of light house labs warranted concerns over the quality of testing and results as they were not held against the usual strict regulation and accreditation of accredited UK laboratories, and mostly did not have HCPC registered professionals performing the tests (96). The pandemic highlighted some poor laboratory practice to the eyes of the public, as televised by BBC panorama when an undercover reporter shared her experience of working in a light house lab, followed by later reports of false negative COVID-19 PCR results being sent to thousands of people (96). This may have unfortunately diminished the respect of highly trained and quality conscious professionals who continuously demonstrate their competence through a variety of means including completing accredited degrees, work-based competencies, and uphold a professional commitment to lifelong learning. It was not until the accumulation of this poor practice was highlighted that the established biomedical science workforce was seen, and registered experts were allowed to engage with the light house laboratory COVID-19 testing (97).

### The IBMS 2022 Strategy

As the professional body for Biomedical Science, the IBMS aim to harness this new platform, as demonstrated in their 2022 strategy (87). Now that the importance of the profession and workforce has been realised, they have been able to source more funding and contribute to policy decisions more than before, and they want to ensure it remains this way. They are actively engaging with parliament through a variety of means to ensure the professions expert opinions will shape future Healthcare decisions (98). They are seeking to improve development opportunities for staff, remove current barriers to HCPC registration, maintain the workforce’s new high profile and extend their global reach and collaborations. One of the barriers to HCPC registration is the availability of non-accredited Biomedical Sciences degrees. Educating students who are keen to enter the Biomedical Science workforce on appropriate degrees and entry routes will aid uptake of graduates with the required skills and competencies. Furthermore, the IBMS aimed to source funding for non-accredited degree assessments for those graduates wanting to enter the workforce but who will need to complete top up modules to allow for registration. This was successfully achieved in March 2022 as funding from NHS England became available. Furthermore, additional funding was released by Health Education England (HEE) to support the recruitment and training of Level 2, 4 and 6 Healthcare Science apprentices in efforts to develop the diagnostic workforce (99). The IBMS aim to work closely with HEIs and industry to support increased workplace opportunities, in addition to improving development opportunities at all grade levels by expanding their e-learning platform and qualifications. This includes movement away from discipline specific training to modular topics to improve skills mix and flexibility to meet service needs. This extends beyond scientific based learning and includes leadership and management skills to give members access to more senior roles. The IBMS also aim to expand their provision of advanced practice qualifications to more disciplines, as this is largely unequal, e.g., well developed in Histology but less so in other disciplines. These objectives echo the desires of laboratory professionals wishing to develop their careers (13). To improve public knowledge of the role of Biomedical Science and the importance of the workforce, the IBMS has also increased efforts to promote the profession in the media (100) and are supporting members to engage more with the media (101). The IBMS are also commissioning PhD and research initiatives to address the lack of published resources regarding the profession (87) and supporting the implementation of Practice Educators in Healthcare Science, discussed previously (93, 102). Through this new direction of the IBMS strategy, a more positive outcome in professional recognition is predicted, and other countries have similar aims, as demonstrated by a statement from the American Society for Clinical Chemistry (103). The pandemic challenges have provided the workforce with an opportunity to display their importance, and previous deficiencies in funding and investment are being addressed that will ultimately allow for a better workforce to be built in the future.

### Impact on Mental Health and Wellbeing

Mental health and wellbeing can impact training and assessment, owing to the difficulty in completing the necessary tasks and impeded motivation when individuals are negatively impacted psychologically (14–18). With limited resources published on the psychological impact the pandemic has had on laboratory staff, the scale of this is not fully understood (12). However, research regarding students, healthcare staff and the public suggests the pandemic has had a large impact and further research and support will be required. Students are known to be a vulnerable group when it comes to mental health and wellbeing, thus the additional impact of the pandemic has warranted attention (8, 9, 10, 14). A study from Saudi Arabia (104) reported medical students that quarantined for 2 weeks became detached from their peers and family and spent less time studying. They postulate these psychological effects of lockdown could be worsened with time and suggest further research is required. Furthermore, a study in Malaysia (14) found healthcare science students are particularly vulnerable due to the complexity of their training. This could be further exacerbated by the barriers to HCPC registration seen in the United Kingdom. For example, students with non-accredited degrees may find after 3–4 years of studying they need to enrol in further top up modules to be able to become a Biomedical Scientist, in addition to completing the IBMS CoC when placement/trainee posts are limited. A non-accredited degree assessment by the IBMS also costs money and the top up modules can be expensive. Thus, it is promising that the IBMS strategy wants to invest funding to support such students to HCPC registration (87). Furthermore, placements are often unpaid, and during the pandemic there were fewer hospitality jobs for students who may normally be able to make some money whilst studying/undergoing placement. Financial difficulties can worsen mental health and invoke stress which can further impact one’s ability to learn/train effectively (14). As employers have recognised the benefits of remote working in providing flexibility for staff and improving environmental sustainability, many organisations are likely to make permanent changes to the way they operate. Thus, learning online and instilling self-efficiency skills into students can better prepare them for the working life of the future. To address challenges such as finding a work/home balance and emotional wellbeing, it has been suggested this can be incorporated into university curriculum to further prepare students (71).

NHS support for staff mental health and wellbeing was expanded during the pandemic (105), however a study in Scotland (3) found that barriers prevented their use for many staff. Although they focussed on front line staff, the key barriers highlighted are likely the same for laboratory workers, e.g., heavy workload, low staffing, and a fear of being judged (3, 110). They suggest it’s not enough just to have support resources in place, but organisational plans to allow staff to access resources are required. A study in Singapore (12) highlights the lack of published data on the psychological impact the pandemic has had on laboratory professionals; in comparison to patient facing professionals such as nurses and doctors. Laboratory staff of various grades/experience participated in an online questionnaire to identify levels of anxiety, fear, depression, and physical symptoms such as loss of appetite, reduced sleep quality and exhaustion. Of the 103 responders working with high-risk samples during the pandemic (25th May 2020–8th June 2020), 62.1% expressed mild-severe depression, 53.4% expressed anxiety, and 55.3% generated a moderate-intense fear score. Statistical analysis also found a significant correlation between increased depression scores and the physical symptoms mentioned above (<*p* = 0.05). The authors acknowledge limitations to the study, such as an inability to cross reference medical history and the participants had to conduct the test without supervision due to social distancing. Thus, it is worth noting that whether participants had a history of mental health prior to the pandemic was not fully considered. In addition, the survey results cannot be compared to pre-pandemic scores, preventing solid conclusions. Participant variables whilst completing the survey, such as interpretation of the questions, could also not be moderated. Furthermore, the participants were largely from a blood science background. With Haematology, Blood Transfusion and Chemistry accounting for 81.2% of responses. Whilst a mere 5.7% of responders were based in Microbiology and Serology, which experienced the most dramatic change to working practices (13). Nevertheless, only 65% of responders were aware of support programmes in place, urging more needs to be done in signposting support. The limitations of the study, such as the inability to compare pandemic with pre-pandemic data further highlight the lack of published information regarding the clinical laboratory workforce.

Although there are limitations to the Singapore study, data from the Office for National Statistics Opinions and Lifestyle Survey suggests increased levels of depression in British adults during the pandemic, in comparison to pre-pandemic statistics (106, 107, 108). Albeit showing a generalised impact on mental health and wellbeing on the adult population, rather than on laboratory professionals. Respondents were considered to be experiencing symptoms when achieving a score of 10 or more on the NHS Clinical Depression 8 item score, which generates a score of 0–20. A score >10 is classified as moderate-severe depression. The June 2020 survey is of particular interest as the same group of individuals were interviewed, allowing comparison of their pre-pandemic (July 2019–March 2020) and pandemic depression scores. The report (106) highlighted 12.9% of respondents went from a pre-pandemic depression score <10 to a score >10 during the pandemic. Furthermore, of the 19.2% responders that reported some form of depression (score >10), 80% stated their wellbeing had been affected by the pandemic, with 84.9% reporting feelings of stress and/or anxiety (105). Although no details regarding professional/job status were collected, sickness rate data from NHS Digital suggests that healthcare professionals are vulnerable to mental health difficulties, demonstrated by this repeatedly being the most common cause of absence for NHS employees (18). However, this is generated from NHS Electronic Staff Record (ESR) data, thus largely depends on NHS Trust’s self-reporting, and has many limitations, e.g., variations in how Trust’s report their absence rates, under reporting, or not reporting reasons for illness.

A group particularly vulnerable to mental health and wellbeing impacts are apprentice trainee Biomedical Scientists as they would face an accumulation of both student and employee challenges, worsened by the pandemic, with a hectic academic and laboratory workload, in addition to their personal lives. They are often required to invest a large proportion of their personal time to catching up with assignments, which leaves little time for rest, increasing their risk of burnout (109). With the increased uptake of apprentices into Biomedical science, also indicated in the NHS Diagnostics and Recovery plan (86), further research, resources, and effective signposting to support apprentices is warranted. The recognition of the impact of mental health and wellbeing has been recognised through its inclusion in the updated HCPC standards of proficiency for Biomedical Scientists summarised in Table 1 (66, 111).

## Conclusion

A realisation of this review is that the impact of the pandemic on training and assessment in the clinical laboratory is multifaceted and cannot simply be summarised. Every level of trainee, from undergraduate, apprentice, staff pursuing further education and those looking to develop their practice, experienced unique challenges. In addition, the impact on different pathology disciplines varied enormously, however all experienced a very different way of working and adapted to novel and stressful situations. Effective workplace planning will require reliable data on the current state of the clinical laboratory workforce (85, 86). Strategies moving forward should address the long-standing concerns regarding staffing and investment in all disciplines relevant to future workloads and testing demands, not only those that were hit worst during the pandemic. Collaboration between HEIs and industry are vital to ensure training is fit for purpose from the very beginning. A common theme throughout this review is that there is limited research published regarding the impact of major change on the clinical laboratory workforce in multiple areas, including their training and development. With the vital contribution this group has on the patient pathway and in national crises, as highlighted by the pandemic, this warrants further attention. Subsequent research is required in relation to the clinical laboratory workforce, barriers to their training, and how the service can be shaped to provide better care for patients.

The outlook is promising as demonstrated by the IBMS corporate strategic plan (87). HEIs are innovating their teaching practices, learning from the transition to ERT, which provided many insights and opportunities that can be harnessed to improve the quality of teaching for the future. A fundamental impact of the pandemic on clinical laboratory training and assessment is highlighted through the recognition of the importance of a quality service for patients and in developing a workforce that is fit to respond to changing patient needs and future crises. Despite the challenges imposed by the pandemic, pathology departments have maintained a quality service and implemented innovative practices that will have a long-lasting impact on the quality of care for the future. Ultimately, this review has aimed to highlight the plethora of challenges faced by the clinical laboratory workforce, pre- and post-pandemic, with special attention on the impact of training at all levels. This may serve as a starting point for what will inevitably be a growing area of research for the future as a result of the workforce’s newfound visibility and subsequent recognition of the vital contribution they make to healthcare and wider society. Furthermore, the 2023 revised QAA benchmark statement for Biomedical Sciences will ensure key knowledge, skills and competences including equality, diversity and inclusion, resilience, leadership and sustainability, will become embedded into higher education courses in the near future (68, 112).

**FIGURE 4:**
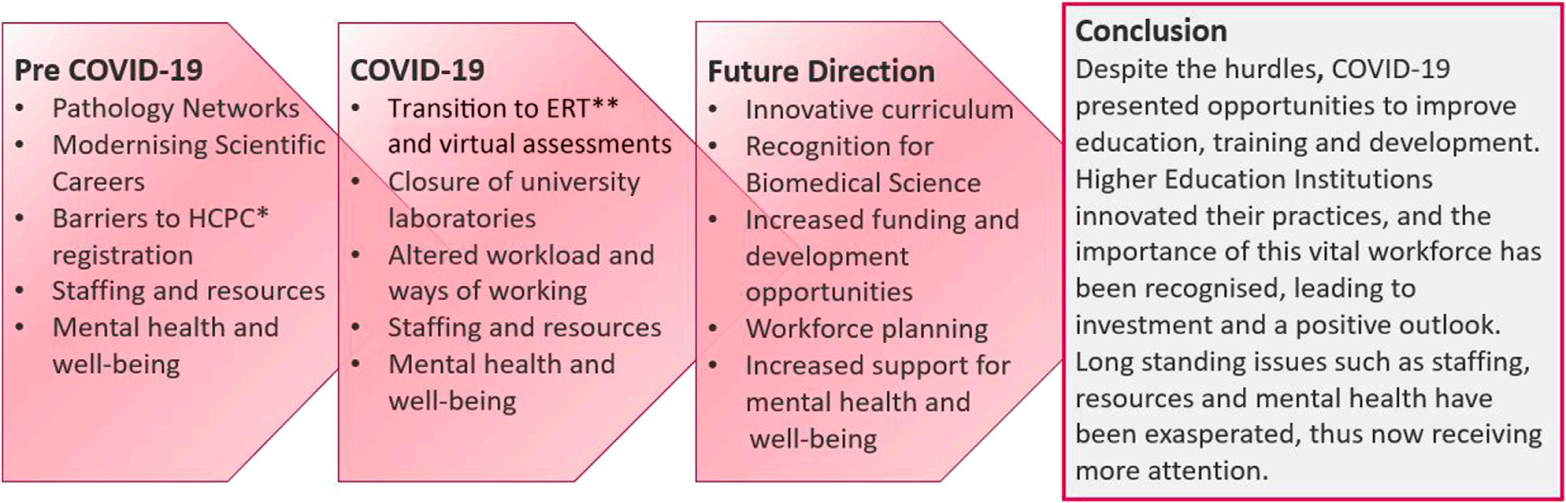
Summary of key challenges pre COVID-19, during COVID-19, and the future outlook for education, training and progression in clinical laboratories. *Health and Care Professions Council **Emergency Remote Teaching.

A summary of the main findings from this review can be found in Figure 4, with final recommendations in Table 4.

**TABLE 4:** Recommendations for improvements to future education, training and professional development of the clinical laboratory workforce.

Education	Higher Education Institutions to consider:
	• the specific needs of their students to personalise learning and share best practice
	• investing in digital technology; including development of staff and student competency
	• implementation of skills for online working, self-efficiency and maintaining a healthy work/home balance into the curriculum as more employers move to remote working
Training	Consider alternative and simulated placement opportunities to expand placement provision
	Emphasise and support skills mix in development opportunities to promote a flexible workforce
	Close collaboration between universities and industry required to ensure education and training is fit for purpose
Assessment	Consider alternative research projects—alternatives to laboratory-based projects include; Bioinformatics/big data; computational modelling; simulation evaluations; systematic reviews containing meta-analysis; surveys/focus groups and educational development evaluations
Workplace Practice	Utilisation of available funding to support education development
	Prioritisation and investment in training support and provision
	Consideration of training needs to be embedded into workforce planning and recruitment as Continual Professional Development is a key requirement for maintaining Health and Care Professions Council registration
Mental Health	Encourage self-awareness and reduction of associated stigma
	Clear signposting of the support available and organisational plans to remove barriers to accessing these
Public Perception	Promote student and employee public engagement and outreach activities

## Limitations

Limited primary resources available in this area, as identified by review, requires further research.
